# Supplementary material for: Modeling glioblastoma heterogeneity as a dynamic network of cell states
Source: Mol Syst Biol. 2021 Sep 16;17(9):e10105. doi: 10.15252/msb.202010105 (PMC8444284; doi:10.15252/msb.202010105)
Supplement: Supplementary file 6 — Source Data for Figure 5 [file MSB-17-e10105-s004.zip › Figure5A_sourcedata/GSEA_3071/hallmarks_stateIV.GseaPreranked.1623742551702/HALLMARK_MTORC1_SIGNALING.html]

Details for gene set HALLMARK\_MTORC1\_SIGNALING[GSEA]

|  || Dataset | state53071 |
| Phenotype | NoPhenotypeAvailable |
| Upregulated in class | na\_neg |
| GeneSet | HALLMARK\_MTORC1\_SIGNALING |
| Enrichment Score (ES) | -0.39285046 |
| Normalized Enrichment Score (NES) | -2.1336603 |
| Nominal p-value | 0.0 |
| FDR q-value | 2.493277E-4 |
| FWER p-Value | 0.002 |
Table: GSEA Results Summary

  

Fig 1: Enrichment plot: HALLMARK\_MTORC1\_SIGNALING      
 Profile of the Running ES Score & Positions of GeneSet Members on the Rank Ordered List

  

| PROBE | GENE SYMBOL | GENE\_TITLE | RANK IN GENE LIST | RANK METRIC SCORE | RUNNING ES | CORE ENRICHMENT || 1 | CTH |  |  | 60 | 1.774 | 0.0101 | No |
| 2 | STC1 |  |  | 97 | 1.547 | 0.0221 | No |
| 3 | TM7SF2 |  |  | 113 | 1.500 | 0.0375 | No |
| 4 | CXCR4 |  |  | 197 | 1.269 | 0.0370 | No |
| 5 | BTG2 |  |  | 200 | 1.260 | 0.0520 | No |
| 6 | CD9 |  |  | 288 | 1.062 | 0.0482 | No |
| 7 | ELOVL6 |  |  | 437 | 0.867 | 0.0302 | No |
| 8 | IDH1 |  |  | 469 | 0.842 | 0.0345 | No |
| 9 | FADS2 |  |  | 675 | 0.674 | 0.0031 | No |
| 10 | PIK3R3 |  |  | 852 | 0.578 | -0.0239 | No |
| 11 | MTHFD2L |  |  | 1122 | 0.486 | -0.0700 | No |
| 12 | ACSL3 |  |  | 1159 | 0.477 | -0.0711 | No |
| 13 | GSK3B |  |  | 1227 | 0.457 | -0.0785 | No |
| 14 | INSIG1 |  |  | 1317 | 0.436 | -0.0903 | No |
| 15 | SLC6A6 |  |  | 1684 | 0.353 | -0.1568 | No |
| 16 | TFRC |  |  | 1750 | 0.339 | -0.1652 | No |
| 17 | VLDLR |  |  | 1866 | 0.319 | -0.1836 | No |
| 18 | MLLT11 |  |  | 1932 | 0.308 | -0.1924 | No |
| 19 | HMGCS1 |  |  | 1974 | 0.302 | -0.1966 | No |
| 20 | SLC1A4 |  |  | 2350 | -0.252 | -0.2660 | No |
| 21 | CYB5B |  |  | 2361 | -0.254 | -0.2648 | No |
| 22 | IMMT |  |  | 2380 | -0.256 | -0.2652 | No |
| 23 | TCEA1 |  |  | 2385 | -0.256 | -0.2628 | No |
| 24 | NFKBIB |  |  | 2404 | -0.259 | -0.2631 | No |
| 25 | PITPNB |  |  | 2533 | -0.273 | -0.2846 | No |
| 26 | NUFIP1 |  |  | 2543 | -0.274 | -0.2830 | No |
| 27 | PPA1 |  |  | 2551 | -0.275 | -0.2809 | No |
| 28 | PSMD12 |  |  | 2609 | -0.284 | -0.2885 | No |
| 29 | LGMN |  |  | 2610 | -0.284 | -0.2850 | No |
| 30 | CDC25A |  |  | 2753 | -0.301 | -0.3088 | No |
| 31 | PDK1 |  |  | 2754 | -0.301 | -0.3051 | No |
| 32 | ABCF2 |  |  | 2767 | -0.302 | -0.3038 | No |
| 33 | GBE1 |  |  | 2881 | -0.317 | -0.3217 | No |
| 34 | PSMC4 |  |  | 2891 | -0.318 | -0.3196 | No |
| 35 | USO1 |  |  | 2936 | -0.323 | -0.3241 | No |
| 36 | GSR |  |  | 3021 | -0.335 | -0.3363 | No |
| 37 | NUP205 |  |  | 3125 | -0.350 | -0.3519 | No |
| 38 | RPA1 |  |  | 3127 | -0.350 | -0.3478 | No |
| 39 | SRD5A1 |  |  | 3135 | -0.352 | -0.3449 | No |
| 40 | PSMC2 |  |  | 3152 | -0.353 | -0.3437 | No |
| 41 | PSMD14 |  |  | 3196 | -0.359 | -0.3476 | No |
| 42 | MCM2 |  |  | 3240 | -0.366 | -0.3514 | No |
| 43 | PGK1 |  |  | 3257 | -0.369 | -0.3500 | No |
| 44 | PSMD13 |  |  | 3348 | -0.381 | -0.3627 | No |
| 45 | P4HA1 |  |  | 3355 | -0.382 | -0.3592 | No |
| 46 | STIP1 |  |  | 3427 | -0.395 | -0.3681 | No |
| 47 | ATP6V1D |  |  | 3441 | -0.396 | -0.3658 | No |
| 48 | PLOD2 |  |  | 3453 | -0.400 | -0.3630 | No |
| 49 | ETF1 |  |  | 3503 | -0.410 | -0.3675 | No |
| 50 | GTF2H1 |  |  | 3539 | -0.417 | -0.3691 | No |
| 51 | HSPA5 |  |  | 3555 | -0.419 | -0.3669 | No |
| 52 | IFRD1 |  |  | 3599 | -0.429 | -0.3700 | No |
| 53 | ITGB2 |  |  | 3649 | -0.435 | -0.3741 | No |
| 54 | MAP2K3 |  |  | 3674 | -0.439 | -0.3734 | No |
| 55 | YKT6 |  |  | 3742 | -0.454 | -0.3808 | No |
| 56 | CCNF |  |  | 3755 | -0.456 | -0.3776 | No |
| 57 | TUBG1 |  |  | 3775 | -0.460 | -0.3756 | No |
| 58 | PPIA |  |  | 3778 | -0.461 | -0.3703 | No |
| 59 | STARD4 |  |  | 3785 | -0.463 | -0.3658 | No |
| 60 | PPP1R15A |  |  | 3793 | -0.465 | -0.3615 | No |
| 61 | HSPD1 |  |  | 3842 | -0.474 | -0.3650 | No |
| 62 | POLR3G |  |  | 3979 | -0.506 | -0.3851 | No |
| 63 | ATP5MC1 |  |  | 4020 | -0.518 | -0.3865 | Yes |
| 64 | TOMM40 |  |  | 4030 | -0.520 | -0.3819 | Yes |
| 65 | PNO1 |  |  | 4044 | -0.524 | -0.3780 | Yes |
| 66 | SDF2L1 |  |  | 4049 | -0.525 | -0.3723 | Yes |
| 67 | GOT1 |  |  | 4102 | -0.541 | -0.3758 | Yes |
| 68 | CACYBP |  |  | 4109 | -0.542 | -0.3703 | Yes |
| 69 | TPI1 |  |  | 4134 | -0.550 | -0.3682 | Yes |
| 70 | NUPR1 |  |  | 4156 | -0.556 | -0.3655 | Yes |
| 71 | HPRT1 |  |  | 4179 | -0.562 | -0.3629 | Yes |
| 72 | PLK1 |  |  | 4205 | -0.569 | -0.3607 | Yes |
| 73 | BUB1 |  |  | 4246 | -0.578 | -0.3614 | Yes |
| 74 | EEF1E1 |  |  | 4257 | -0.583 | -0.3562 | Yes |
| 75 | ARPC5L |  |  | 4269 | -0.588 | -0.3511 | Yes |
| 76 | BCAT1 |  |  | 4272 | -0.590 | -0.3443 | Yes |
| 77 | IGFBP5 |  |  | 4280 | -0.592 | -0.3384 | Yes |
| 78 | CCT6A |  |  | 4304 | -0.598 | -0.3355 | Yes |
| 79 | NFIL3 |  |  | 4309 | -0.600 | -0.3290 | Yes |
| 80 | CYP51A1 |  |  | 4343 | -0.612 | -0.3279 | Yes |
| 81 | GLRX |  |  | 4374 | -0.626 | -0.3260 | Yes |
| 82 | TXNRD1 |  |  | 4382 | -0.629 | -0.3197 | Yes |
| 83 | HSPA9 |  |  | 4410 | -0.639 | -0.3171 | Yes |
| 84 | PNP |  |  | 4417 | -0.641 | -0.3104 | Yes |
| 85 | ERO1A |  |  | 4434 | -0.647 | -0.3056 | Yes |
| 86 | DDX39A |  |  | 4467 | -0.661 | -0.3037 | Yes |
| 87 | UCHL5 |  |  | 4510 | -0.685 | -0.3034 | Yes |
| 88 | DHFR |  |  | 4517 | -0.688 | -0.2962 | Yes |
| 89 | SLC7A5 |  |  | 4544 | -0.701 | -0.2926 | Yes |
| 90 | NAMPT |  |  | 4551 | -0.705 | -0.2851 | Yes |
| 91 | EBP |  |  | 4571 | -0.718 | -0.2800 | Yes |
| 92 | LDHA |  |  | 4574 | -0.722 | -0.2716 | Yes |
| 93 | SLC2A3 |  |  | 4629 | -0.754 | -0.2728 | Yes |
| 94 | SEC11A |  |  | 4647 | -0.763 | -0.2668 | Yes |
| 95 | PSMA3 |  |  | 4667 | -0.773 | -0.2610 | Yes |
| 96 | ACTR3 |  |  | 4696 | -0.795 | -0.2567 | Yes |
| 97 | G6PD |  |  | 4698 | -0.796 | -0.2471 | Yes |
| 98 | SHMT2 |  |  | 4737 | -0.820 | -0.2444 | Yes |
| 99 | PRDX1 |  |  | 4783 | -0.848 | -0.2428 | Yes |
| 100 | SERPINH1 |  |  | 4829 | -0.895 | -0.2405 | Yes |
| 101 | EIF2S2 |  |  | 4853 | -0.915 | -0.2338 | Yes |
| 102 | PSAT1 |  |  | 4867 | -0.932 | -0.2249 | Yes |
| 103 | AURKA |  |  | 4906 | -0.973 | -0.2203 | Yes |
| 104 | SQSTM1 |  |  | 4929 | -0.994 | -0.2124 | Yes |
| 105 | MCM4 |  |  | 4947 | -1.011 | -0.2033 | Yes |
| 106 | SLC2A1 |  |  | 4949 | -1.018 | -0.1911 | Yes |
| 107 | ME1 |  |  | 4950 | -1.019 | -0.1786 | Yes |
| 108 | PSMG1 |  |  | 4955 | -1.025 | -0.1669 | Yes |
| 109 | SYTL2 |  |  | 4992 | -1.079 | -0.1606 | Yes |
| 110 | TUBA4A |  |  | 5010 | -1.111 | -0.1503 | Yes |
| 111 | XBP1 |  |  | 5032 | -1.162 | -0.1402 | Yes |
| 112 | MTHFD2 |  |  | 5101 | -1.297 | -0.1374 | Yes |
| 113 | ENO1 |  |  | 5146 | -1.410 | -0.1287 | Yes |
| 114 | SLC1A5 |  |  | 5154 | -1.444 | -0.1124 | Yes |
| 115 | TRIB3 |  |  | 5168 | -1.526 | -0.0962 | Yes |
| 116 | PHGDH |  |  | 5170 | -1.528 | -0.0777 | Yes |
| 117 | DDIT3 |  |  | 5229 | -1.853 | -0.0663 | Yes |
| 118 | RRM2 |  |  | 5232 | -1.875 | -0.0437 | Yes |
| 119 | SLC7A11 |  |  | 5257 | -2.165 | -0.0219 | Yes |
| 120 | SLC9A3R1 |  |  | 5260 | -2.295 | 0.0058 | Yes |
Table: GSEA details [plain text format]

  

Fig 2: HALLMARK\_MTORC1\_SIGNALING: Random ES distribution      
 Gene set null distribution of ES for **HALLMARK\_MTORC1\_SIGNALING**
